# The Connectome and Chemo-Connectome Databases for Mice Brain Connection Analysis

**DOI:** 10.3389/fnana.2022.886925

**Published:** 2022-06-09

**Authors:** Yang Wang, Zhixiang Liu, Da Sun, Leqiang Sun, Gang Cao, Jinxia Dai

**Affiliations:** ^1^State Key Laboratory of Agricultural Microbiology, Huazhong Agricultural University, Wuhan, China; ^2^College of Veterinary Medicine, Huazhong Agricultural University, Wuhan, China; ^3^Britton Chance Center for Biomedical Photonics, Wuhan National Laboratory for Optoelectronics, Huazhong University of Science and Technology, Wuhan, China; ^4^College of Veterinary Medicine, China Agricultural University, Beijing, China; ^5^Biomedical Center, Huazhong Agricultural University, Wuhan, China

**Keywords:** connectome, chemo-connectome, database, mesoscale connection, monoaminergic nuclei

## Abstract

The various brain functions rely on the intricate connection networks and certain molecular characteristics of neurons in the brain. However, the databases for the mouse brain connectome and chemo-connectome are still inadequate, hindering the brain circuital and functional analysis. Here, we created mice brain connectome and chemo-connectome databases based on mouse brain projection data of 295 non-overlapping brain areas and *in situ* hybridization (ISH) data of 50 representative neurotransmission-related genes from the Allen Brain Institute. Based on this connectome and chemo-connectome databases, functional connection patterns and detailed chemo-connectome for monoaminergic nuclei were analyzed and visualized. These databases will aid in the comprehensive research of the mouse connectome and chemo-connectome in the whole brain and serve as a convenient resource for systematic analysis of the brain connection and function.

## Introduction

The functions of the brain rely on its complex and delicate networks, in which certain neural connections are responsible for specific brain functions that include sensation, movement, cognition, and emotion. Mapping neural circuits’ organization and their function are fundamental and crucial for understanding our brain. Especially, delineating the connectome, referring to the connection networks in the whole brain (Sporns et al., 2005), is challenging but essential for neuroscience research. Meanwhile, the chemical characteristics of neurons that include neurotransmitters, neuromodulators, and transmitter receptors determine neuron communication during neural transmission within brain networks (Cooper et al., 2003). Thus, the chemo-connectome of which connectome contains the neurotransmitters, neuromodulators, and receptors information is equally important for connectome resolution (Deng et al., 2019). These connection networks and chemical molecule networks are the basis for the brain to perform various functions (Luo et al., 2018).

According to the scale of neural connectivity, neural circuit delineation can be performed at four levels: the macroscale connection between brain regions, the mesoscale connection between different types of neurons, the microscale connection between individual neurons, and the nanoscale connection at the synaptic level (Swanson and Lichtman, 2016). Different levels interpret distinct resolution connections, which meet certain functional studies. To date, the microscale connectome is only completed in a few organisms that include nematode *Caenorhabditis elegans* (*C. elegans*) and *Pristionchus pacificus*, as well as partial neural circuits of mice (such as the retina and portion of neocortex) and *Drosophila* (such as visual system, mushroom body, locomotion, and larval escape response) (Zingg et al., 2014; Robie et al., 2017; Cook et al., 2019). Due to the complex organization of the mammalian brain (hundreds of nuclei, millions of neurons, and tens of billions of connections), the brain connectome studies are mainly performed at the macroscale and mesoscale levels.

In the case of mice, with the anterograde and retrograde chemical or viral tracers, certain output and input neural networks and their function are resolved (Beier et al., 2015; Schwarz et al., 2015). Allen Brain Institute provided mesoscale input and output connection database for almost all key brain nuclei of mice, which greatly facilitated brain functional research (Oh et al., 2014; Kuan et al., 2015; Wang et al., 2020). By collecting, sorting, and analyzing mice brain connection data and *in situ* hybridization (ISH) data from Allen Brain Institute and other reported data, the connectome and chemo-connectome for certain brain functions in mice whole brain were obtained (Zeng et al., 2015; Swanson et al., 2016, 2017, 2018). However, it really needs extensive work to get interested connectome even chemo-connectome through collecting the huge corresponding data. A convenient database for easy exporting the connectome and chemo-connectome for certain brain function is desperately needed.

Therefore, in the present study, we aimed to create connectome and chemo-connectome databases for neural research on mice brain. Through summarizing mouse brain connectivity data from Allen Brain Institute and similarity processing analysis, we obtained the connection matrix of 295 non-overlapping brain areas and the projection similarity matrix. Further, after processing the ISH data from Allen Brain Institute, the whole-brain distribution matrix of 50 neurotransmission-related genes that include transmitters and transmitter receptor is finally acquired. *Via* this connectome and chemo-connectome databases, certain functional connection patterns and detailed chemo-connectome for monoaminergic nuclei were visualized. Our connectome and chemo-connectome databases will help to facilitate a comprehensive connection analysis of the mouse whole brain, contributing to a better understating of brain functions.

## Materials and Methods

### Mouse Brain Connectivity Data Processing for the Connectome Database

**FIGURE 1:**
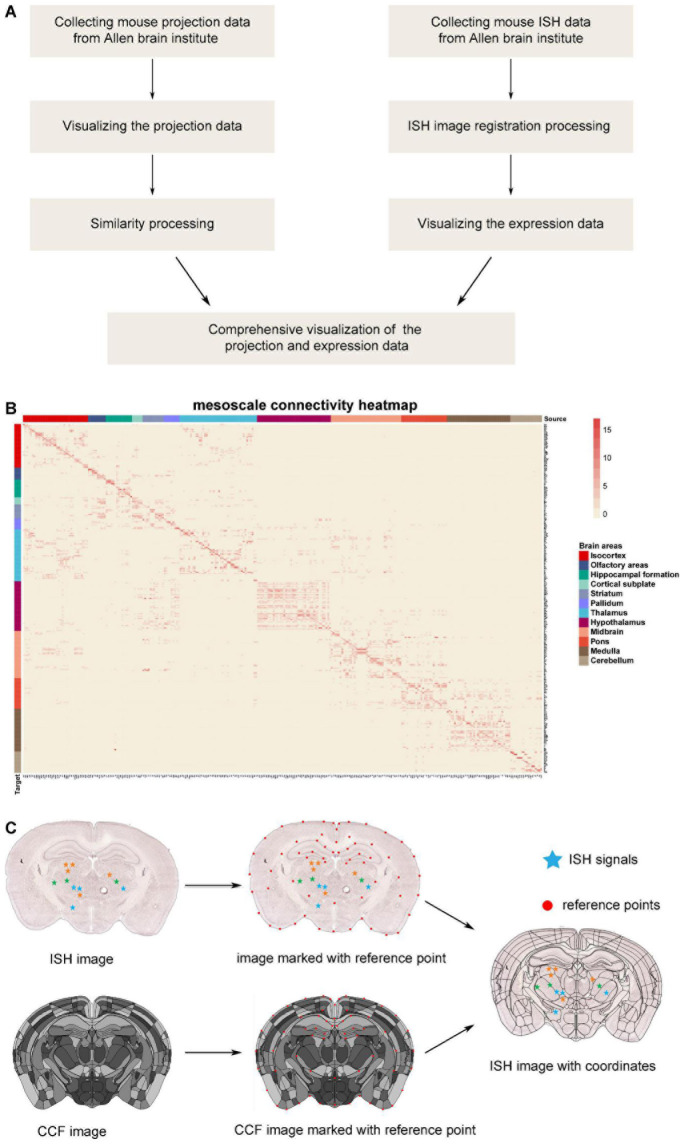
The database processing flow for connectome and chemo-connectome. **(A)** Data processing flowchart for creating connectome and chemo-connectome databases. **(B)** Heatmap of the whole-brain connectivity at mesoscale level. The full names of abbreviations for all the source and target areas are listed in Supplementary Table 6. **(C)** Diagram of the ISH data processing. ISH, *in situ* hybridization; CCF, common coordinate framework.

Collection of projection data from Allen Brain Institute: firstly, 499 sets of output projection data for different brain areas traced by anterograde adeno-associated viruses (AAV) virus in wild-type C57BL/6J mice were downloaded from the Allen Brain Institute (Figure 1A). The projection data are a list of signal intensity (sum of detected pixel intensity/sum of detected pixels) for 294 brain areas. In the case of brain areas with multiple tracing, we took the average value of the projection intensity (total signal intensity divided by the number of repeats). Then, the value of 0.0004 was selected as the projection intensity threshold to ignore the mild projections. Thus, we got the whole-brain connection strength matrix (Supplementary Table 1), and the corresponding connection heatmap is shown in Figure 1B.

Similarity analysis: the connectome data were standardized by the z-score method, then the Pearson’s correlation coefficients between each pair of source areas were calculated based on their projection density toward target areas. The hierarchical clustering, a widely applied method (Harris et al., 2019), was applied to cluster the brain nuclei into groups (data are listed in Supplementary Table 2), and the similarity heatmaps of input and output networks were delineated respectively.

### Mouse Brain *in situ* Hybridization Data Processing for the Chemo-Connectome Database

The ISH images were registered to the standard atlas by the previously reported graphical user interface (GUI) based on the MATLAB platform (Chen et al., 2021). The processing pipeline is illustrated in Figures 1A,C.

In detail, the reference points were manually labeled on both common coordinate framework (CCF) images and ISH images. These points are usually located on the boundaries of brain structures, such as the cortex, ventricles, and the prominent regions with a special texture of ISH signals. To simplify the manual operation, the slice images would be trimmed and rotated. In addition, some points could be added to slice the image automatically depending on the relative position of existing atlas feature points. In some cases, the section plane of brain slice was inconsistent with the ideal coronal plane, which was generally presented in the standard atlas. However, the three-dimensional continuity of CCF made it possible to get a 2D atlas section at any angle. Moreover, with the help of angle rotation tool of GUI in MATLAB, the slant section was registered well. Once the reference point labeling was finished, a non-linear deformation field was calculated with these point pairs by the thin plate spline (TPS) method (Duchon, 2006). Because the pixels representing the ISH signal had been recognized and labeled with different colors indicating distinct expression levels, the coordinates of these pixels were mapped into the corresponding nuclei through the non-linear deformation. Finally, the gene expression density was counted and the chemo-connectome database was organized in Supplementary Table 1.

### Data Visualizing

The pheatmap, circlize, networkD3 package, and the corresponding codes (Supplementary Material 1) in R software were used to draw graphics. The connectivity and gene expression data (Supplementary Table 3) were prepared to generate the heatmap, and the data displayed here were standardized by a *z*-score method for better visualization. The data of the top 50 connection and monoaminergic nuclei connection are stored in Supplementary Table 4. In the graphs of the connection network, the arrows between the nuclei represent the projection direction, and the width of the line represents the projection intensity. For the visualization of the gene expression levels of different nuclei, the data for gene expression density of brain nuclei (Supplementary Table 5) were loaded into the program separately. Finally, the adobe illustrator was used to integrate the nuclei connection and gene expression graphs to perform a comprehensive display of the chemical connection of monoaminergic nuclei groups.

A data storage file brain.mat containing three-dimensional coordinates and connectome data of the 295 nuclei were created, which can be easily processed by MATLAB. Moreover, the script file 3d-brain.m was imported into MATLAB for visualization. Line 30 of the script command for *i* = 1:2,000 was used to display the top 2,000 connection network. According to different purposes, the parameters can be adjusted, and the function-related mesoscale connection data can also be imported into the projectv file for display. The codes of these files are provided in Supplementary Material 2.

## Results

### Overall Display of Mesoscale Connection Network of Mice Whole Brain *via* Connectome Database

**FIGURE 2:**
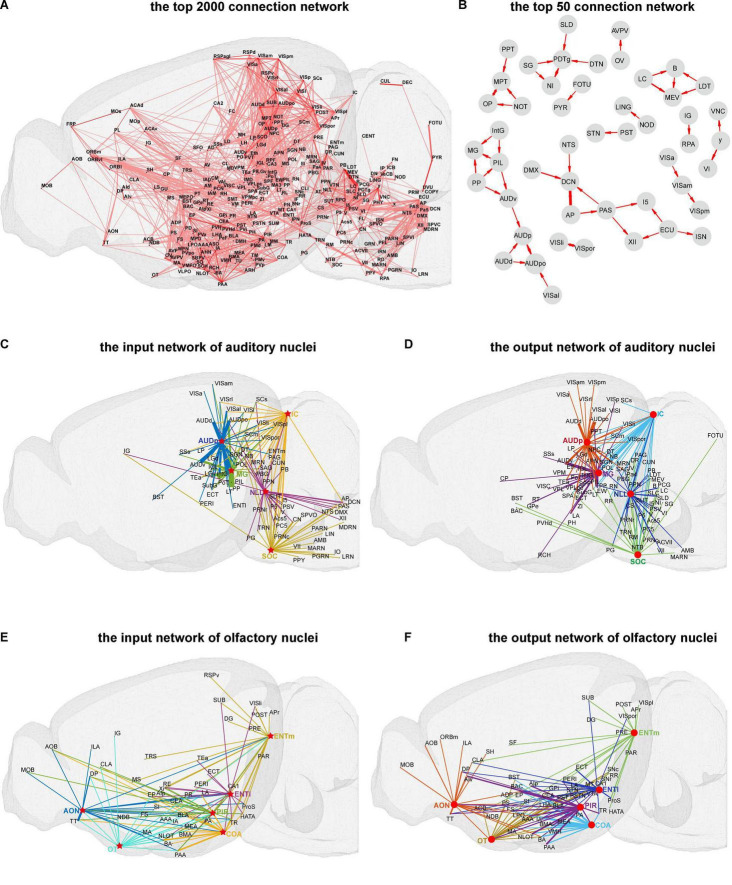
Visualization of the connection network in mice brain. **(A)** The top 2,000 three-dimensional (3D) connection network. **(B)** The top 50 connection networks. **(C,D)** The input and output 3D connection networks of auditory nuclei. **(E,F)** The input and output 3D connection networks of olfactory nuclei. The arrows between the nuclei represent the projection direction, and the width of the line represents the projection intensity. Red circles and stars respectively represent the source and target nuclei in the auditory and olfactory networks.

As described in the methods part, we created the connectome database for mice brain. To obtain an overview of mice brain connectome at the mesoscale level, a three-dimensional illustration of the top 2,000 connections in the whole brain of mice is displayed in Figure 2A. As illustrated, the brain areas are located in isocortex, olfactory areas, hippocampal formation, cortical subplate, striatum, pallidus, thalamus, hypothalamus, midbrain, pons, and medulla form complex high-strength networks with each other. The brain areas in the isocortex are mainly located in the orbital area, medial prefrontal cortex, visual cortex, auditory areas, agranular insular area, somatomotor areas, and retrosplenial areas, which control the processing of various information in the brain and are vital to the survival of mice (Luo, 2016). Further, the top 50 connection networks of the whole brain are shown in Figure 2B. From the top 50 connection networks, we found that most of these brain nuclei belong to the brain stem that includes midbrain, pons, and medulla. Meanwhile, the auditory function-related nuclei [ventral auditory area (AUDv), primary auditory area (AUDp), and dorsal auditory area (AUDd)] and the thalamus nuclei [medial geniculate complex (MG), intermediate geniculate nucleus (IntG), posterior intralaminar thalamic (PIL) nucleus, and peripeduncular (PP) nucleus] form high-strength connection network to the remained brain areas. There are also strong projections between the visual function-related nuclei [laterointermediate area (VISli), postrhinal area (VISpor), anterolateral visual area (VISal), anteromedial visual area (VISam), and posteromedial visual area (VISpm)]. It is of note that the VISal sends projection to the posterior auditory area (AUDpo), suggesting possible signal integration between visual and auditory sensation. Further, the input heatmap and output heatmap, such as visual, auditory, and olfactory functions, of sensory modality are illustrated in Supplementary Figure 1.

### Display of Auditory and Olfactory Function-Related Connection Network

Further, we performed a three-dimensional display of connection networks for certain brain functions. Auditory and olfactory functions are relatively conservative across species, basically important for survival, and also well studied (Butler and Hodos, 2005; Igarashi et al., 2012). Here, the input and output networks of auditory and olfactory function-related nuclei were analyzed (Figures 2C–F). For auditory function, mainly five nuclei, i.e., nucleus of lateral lemniscus (NLL), superior olivary complex (SOC), inferior colliculus (IC), MG, and AUDp, control the auditory information flow in mice central nervous system. The top 30 input and output networks of these five auditory function-related nuclei were illustrated (Figures 2C,D). From the input networks, the major auditory transmission flows from NLL and SOC to IC, then to MG, and finally to AUDp can be observed. At the same time, other cortical and subcortical areas are extensively connected to these five nuclei, such as strong efferent projection from visual cortex sub-areas to AUDp, probably suggesting a convergence between visual and auditory information.

For olfactory function, the top 20 input and output networks of five major nuclei, i.e., anterior olfactory nucleus (AON), olfactory tubercle (OT), piriform area (PIR), cortical amygdala area (COA), and entorhinal area (ENT), are presented in Figures 2E,F. Of the network, AON, COA, and PIR receive projections from the main olfactory bulb (MOB) and accessory olfactory bulb (AOB), and there are mutual projections among the nuclei of AON, OT, COA, PIR, and ENT. We also found that OT emits strong projections to ventromedial hypothalamic nucleus (VMH) and lateral hypothalamic area (LHA), two important nuclei which regulate the feeding behavior (Steffens et al., 1988; Wang et al., 2021).

### Projection Similarity Among Certain Nuclei

**FIGURE 3:**
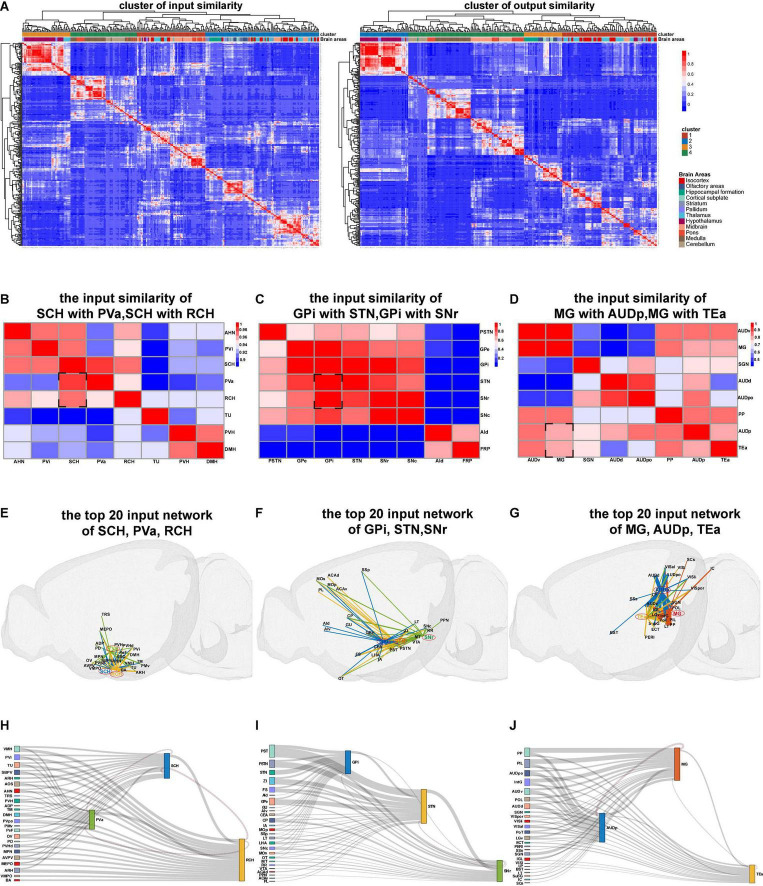
The results of connection similarity processing. **(A)** Heatmaps for input and output similarity clusters of mice whole brain. **(B–D)** Heatmaps of the input similarity nuclei. **(E–G)** The 3D network of input similarity nuclei in panels **(B–D)**. **(H–J)** The Sankey diagram of input similarity nuclei in panels **(B–D)**.

When the nuclei show similar projection patterns, these nuclei may belong to the same functional modality (Butler and Hodos, 2005; Fuster, 2015). Therefore, we next processed similarity analysis based on the whole-brain connectome database according to the input and output similarities of nuclei and finally obtained the input similarity cluster heatmap and output similarity cluster heatmap of mice whole brain (Figure 3A). In the heatmap, the similar projection patterns within 12 brain areas, such as isocortex, hippocampal formation, and midbrain, were directly visualized. Meanwhile, similar projection patterns across 12 brain areas were also presented. Three sets of brain nuclei with a similar input network from the input similarity cluster heatmap were separately exhibited (Figures 3B–D).

The input similarity of the suprachiasmatic nucleus (SCH) with periventricular hypothalamic nucleus anterior part (PVa) and retrochiasmatic area (RCH) is highly above 0.96 (Figure 3B). We extracted the top 20 input projection data of SCH, PVa, and RCH nuclei to respectively display the three-dimensional connection map and the Sankey diagram (Figures 3E,H). Moreover, we observed that the main input projections to the three hypothalamic nuclei (SCH, PVa, and RCH) came from the adjacent nucleus of the hypothalamus. Meanwhile, from the Sankey diagram, the upstream nuclei of the three nuclei are almost the same.

The input similarity of Globus pallidus internal (GPi) segment with subthalamic nucleus (STN) and the substantia nigra reticular (SNr) part is greater than 0.8 (Figure 3C). Although these nuclei are located in different brain regions, such as pallidus, midbrain, and hypothalamus, they all belong to the basal ganglia (Calabresi et al., 2014; Park et al., 2020). We found the upstream nuclei of them are almost the same (Figures 3F,I).

The MG, AUdp, and temporal association areas (TEas) are functionally related to the transmission and processing of auditory information (Tasaka et al., 2020). From the input similarity cluster heatmap, we found that their projection pattern is also very similar (Figures 3G,J). These similarity analysis data can provide resources for the researchers to study the structure and function of brain nuclei that are similar in evolution and development.

### Chemo-Connectome of Whole Brain and Monoaminergic Nuclei Network

The connection network is the structural basis for the brain to perform various functions, and the transmitters and receptors in the neurons decide the communication information of neural circuit. We further processed and summarized the ISH data from Allen Brain Institute to obtain the chemo-connectome of mice brain networks. The expression matrix of 50 transmission-related genes, such as transmitters (classical inhibitory and excitatory transmitters, monoamines, and neuropeptides) and corresponding receptors in the whole brain, were analyzed.

**FIGURE 4:**
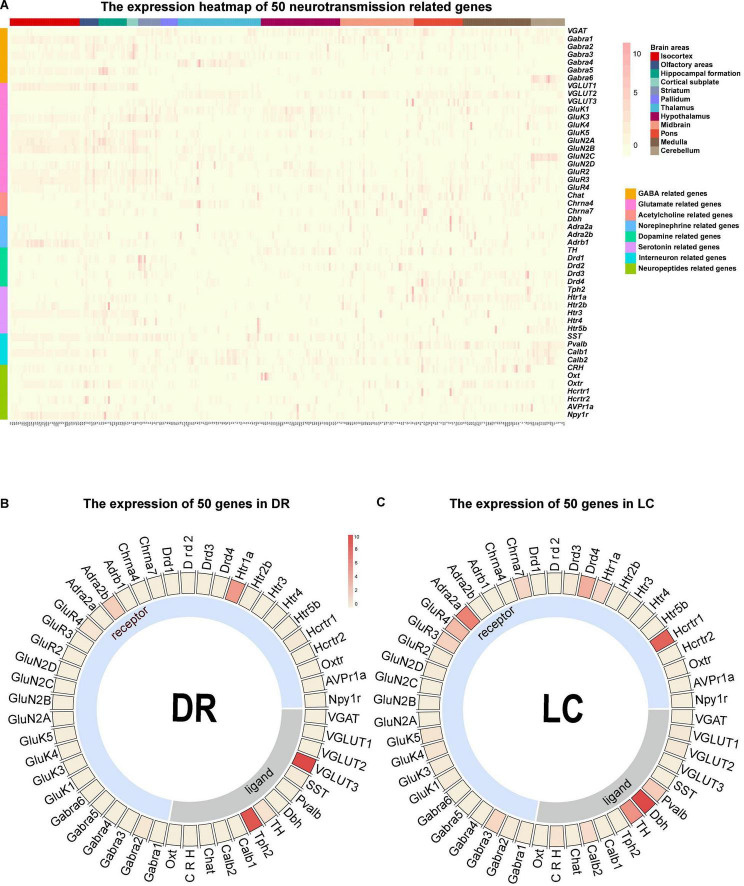
Overall view of the chemo-connectome database. **(A)** Heatmap of the chemo-connectome database. **(B,C)** Circos diagram of neurotransmission- related gene expression in dorsal raphe (DR) and locus coeruleus (LC). The color represents the expression density.

Firstly, we converted the whole-brain expression matrix into a heatmap (Figure 4A). In the dorsal raphe (DR) and the locus coeruleus (LC), the heatmap showed that tryptophan hydroxylase 2 (Tph2, the rate-limiting enzyme for serotonin synthesis) is highly expressed in the DR [the main nucleus of serotonergic neurons (Waider et al., 2011)], and dopamine β hydroxylase (Dbh) is highly expressed in noradrenergic nucleus LC (Itoi and Sugimoto, 2010; Figures 4B,C), which confirmed the reliability of our chemo-connectome database.

**FIGURE 5:**
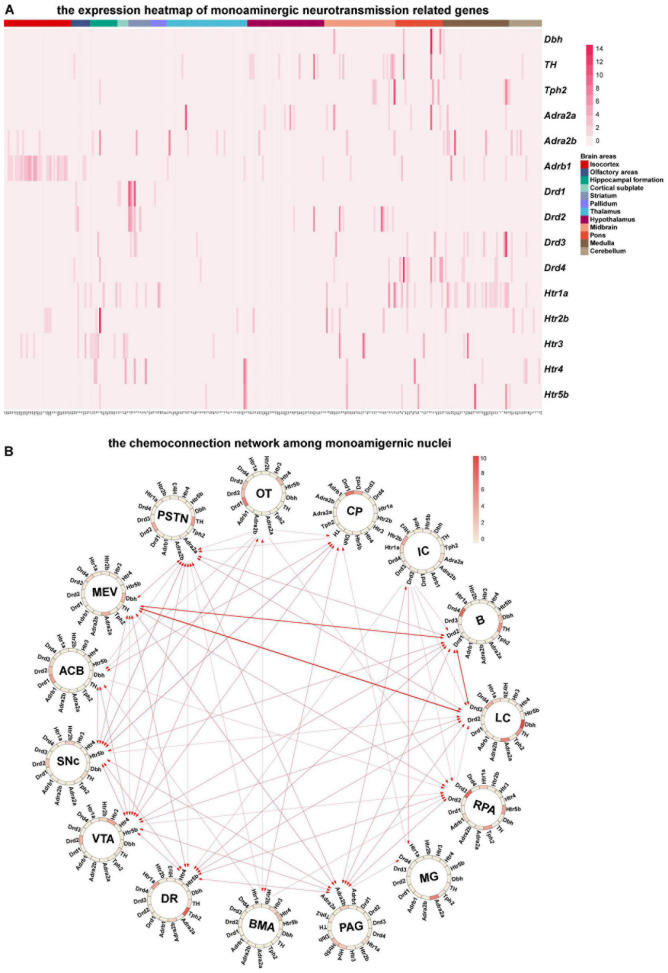
Chemo-connectome of monoaminergic nuclei. **(A)** The expression heatmap of monoaminergic neurotransmission-related genes in mice whole brain. **(B)** The connectivity network and gene expression of monoaminergic nuclei were integrated to generate chemo-connection network. Arrows between the nuclei represent the projection direction, and the width of the line represents the projection intensity. The color in the circos diagram represents the expression density.

Since monoaminergic neurotransmitters (Norepinephrine, dopamine, and serotonin) in the central nervous system are key transmitters involved in the regulation of various essential brain functions, such as arousal and mood (Nutt, 2008), we mainly analyzed the chemo-connectome of the central monoaminergic system through combined ISH data with the connection data (Figures 5A,B). As shown in Figure 5B, three monoaminergic nuclei localized in the hindbrain (locus ceruleus, LC; Barrington’s nucleus, B; nucleus raphe pallidus, RPa) not only connected with each other but also displayed extensive interconnections with other monoaminergic nuclei. It is of note that all of the three nuclei (LC, B, and RPA) displayed high-level expressions of dopamine receptors. The midbrain monoaminergic nuclei dorsal raphe (DR), periaqueductal gray (PAG), ventral tegmental area (VTA), and substantia nigra compact part (SNc), which express different subtypes of dopamine and serotonin receptors, form a complex network within them and also with other monoaminergic nuclei (Figure 5B). Based on the chemo-connectome data, dopamine may play a fundamental role in the function of monoaminergic nuclei.

## Discussion

In neuroscience research, mice are widely employed as model organisms, and various studies on mouse brain structure and function have expanded our knowledge of the brain. In the present study, we first created the mouse brain connectome and chemical connectome databases based on the connectivity and ISH data from the Allen Brain Institute, which achieved our goal of bridging the nuclei connectivity and circuit function. These databases provide a comprehensive perspective to dissect how the brain works and can help researchers easily to realize whole-brain connectivity and certain connection with expression patterns. The degree of similarity in brain nuclei reflects their homology in development or function. Based on connectivity data, we perform similarity processing to obtain the projection similarity data, which can also be used by evolutionists to guide evolutionary analysis. Our work, taken collectively, has the potential to advance the systematic study of the brain.

Due to the limited sources of connectivity and ISH data we used, our mesoscopic connectome and chemo-connectome databases will be improved along with more circuit tracing and gene expression data released, especially the neuron-level connection data, and even microscale level connection data. As for the data analysis method in this study, we firstly selected those brain nuclei with well-known functions and then focused on the local networks in which the selected nucleus served as center nodes. However, this strategy limited the possibility of finding the unreported center brain nuclei and their connection network. Some automatic algorithms could be applied in the future for assessing the characteristic quantity of a brain nucleus network, such as centrality and modularity (van den Heuvel and Hulshoff Pol, 2010).

The study of the mammalian brain connectome and chemo-connectome is still in its early stages. However, based on the success of genomics, we expect that as technology progresses and more researchers enter this field, the study of connectome and chemo-connectome at all scales will be improved, and our understanding of the brain will gradually become evident.

## Data Availability Statement

The original contributions presented in this study are included in the article/Supplementary Material, further inquiries can be directed to the corresponding author.

## Author Contributions

JD and GC conceived and designed the study. DS and LS performed the data collection. YW and ZL performed the data analysis. YW and JD prepared the figures and wrote the manuscript. All authors contributed to the article and approved the submitted version.

## Conflict of Interest

The authors declare that the research was conducted in the absence of any commercial or financial relationships that could be construed as a potential conflict of interest.

## Publisher’s Note

All claims expressed in this article are solely those of the authors and do not necessarily represent those of their affiliated organizations, or those of the publisher, the editors and the reviewers. Any product that may be evaluated in this article, or claim that may be made by its manufacturer, is not guaranteed or endorsed by the publisher.

## Supplementary Material

Supplementary Figure 1 The sensory modality connectivity heatmap. **(A)** The sensory modality input heatmap. **(B)** The sensory modality output heatmap.
