# Supplementary material for: The Connectome and Chemo-Connectome Databases for Mice Brain Connection Analysis
Source: Front Neuroanat. 2022 Jun 9;16:886925. doi: 10.3389/fnana.2022.886925 (PMC9218099; doi:10.3389/fnana.2022.886925)
Supplement: Supplementary file 8 [file Table_6.DOC]

# Abbreviations

| AAA | Anterior amygdalar area | ORBvl | Orbital area, ventrolateral part |
| --- | --- | --- | --- |
| ACAd | Anterior cingulate area, dorsal part | OT | Olfactory tubercle |
| ACAv | Anterior cingulate area, ventral par | OV | Vascular organ of the lamina terminalis |
| ACB | Nucleus accumbens | P5 | Peritrigeminal zone |
| Acs5 | Accessory trigeminal nucleus | PA | Posterior amygdalar nucleus |
| ACVII | Accessory facial motor nucleus | Pa4 | Paratrochlear nucleus |
| AD | Anterodorsal nucleus | Pa5 | Paratrigeminal nucleus |
| ADP | Anterodorsal preoptic nucleus | PAA | Piriform-amygdalar area |
| AHN | Anterior hypothalamic nucleus | PAG | Periaqueductal gray |
| AId | Agranular insular area, dorsal part | PAR | Parasubiculum |
| AIp | Agranular insular area, posterior part | PARN | Parvicellular reticular nucleus |
| AIv | Agranular insular area, ventral part | PAS | Parasolitary nucleus |
| AM | Anteromedial nucleus | PB | Parabrachial nucleus |
| AMB | Nucleus ambiguus | PBG | Parabigeminal nucleus |
| AN | Ansiform lobule | PC5 | Parvicellular motor 5 nucleus |
| AOB | Accessory olfactory bulb | PCG | Pontine central gray |
| AON | Anterior olfactory nucleus | PCN | Paracentral nucleus |
| AP | Area postrema | PD | Posterodorsal preoptic nucleus |
| APN | Anterior pretectal nucleus | PDTg | Posterodorsal tegmental nucleus |
| APr | Area prostriata | PeF | Perifornical nucleus |
| ARH | Arcuate hypothalamic nucleus | PERI | Perirhinal area |
| ASO | Accessory supraoptic group | PF | Parafascicular nucleus |
| AT | Anterior tegmental nucleus | PFL | Paraflocculus |
| AUDd | Dorsal auditory area | PG | Pontine gray |
| AUDp | Primary auditory area | PGRN | Paragigantocellular reticular nucleus |
| AUDpo | Posterior auditory area | PH | Posterior hypothalamic nucleus |
| AUDv | Ventral auditory area | PHY | Perihypoglossal nuclei |
| AV | Anteroventral nucleus of thalamus | PIL | Posterior intralaminar thalamic nucleus |
| AVP | Anteroventral preoptic nucleus | PIR | Piriform area |
| AVPV | Anteroventral periventricular nucleus | PL | Prelimbic area |
| B | Barrington's nucleus | PMd | Dorsal premammillary nucleus |
| BA | Bed nucleus of the accessory olfactory tract | PMv | Ventral premammillary nucleus |
| BAC | Bed nucleus of the anterior commissure | PN | Paranigral nucleus |
| BLA | Basolateral amygdalar nucleus | PO | Posterior complex of the thalamus |
| BMA | Basomedial amygdalar nucleus | POL | Posterior limiting nucleus of the thalamus |
| BST | Bed nuclei of the stria terminalis | POST | Postsubiculum |
| CA1 | Field CA1 | PoT | Posterior triangular thalamic nucleus |
| CA2 | Field CA2 | PP | Peripeduncular nucleus |
| CA3 | Field CA3 | PPN | Posterior pretectal nucleus |
| CEA | Central amygdalar nucleus | PPT | Posterior pretectal nucleus |
| CENT | Central lobule | PPY | Parapyramidal nucleus |
| CL | Central lateral nucleus of the thalamus | PR | Perireunensis nucleus |
| CLA | Claustrum | PRE | Presubiculum |
| CLI | Central linear nucleus raphe | PRM | Paramedian lobule |
| CM | Central medial nucleus of the thalamus | PRNc | Pontine reticular nucleus, caudal part |
| CN | Cochlear nuclei | PRNr | Pontine reticular nucleus |
| COA | Cortical amygdalar area | ProS | Prosubiculum |
| COPY | Copula pyramidis | PS | Parastrial nucleus |
| CP | Caudoputamen | PST | Preparasubthalamic nucleus |
| CS | Superior central nucleus raphe | PSTN | Parasubthalamic nucleus |
| CUL | Culmen | PSV | Principal sensory nucleus of the trigeminal |
| CUN | Cuneiform nucleus | PT | Parataenial nucleus |
| DCN | Dorsal column nuclei | PVa | Periventricular hypothalamic nucleus, anterior part |
| DEC | Declive (VI) | PVH | Paraventricular hypothalamic nucleus |
| DG | Dentate gyrus | PVHd | Paraventricular hypothalamic nucleus, descending division |
| DMH | Dorsomedial nucleus of the hypothalamus | PVi | Periventricular hypothalamic nucleus, intermediate part |
| DMX | Dorsal motor nucleus of the vagus nerve | PVp | Periventricular hypothalamic nucleus, posterior part |
| DN | Dentate nucleus | PVpo | Periventricular hypothalamic nucleus, preoptic part |
| DP | Dorsal peduncular area | PVT | Paraventricular nucleus of the thalamus |
| DR | Dorsal nucleus raphe | PYR | Pyramus (VIII) |
| DT | Dorsal terminal nucleus of the accessory optic tract | RCH | Retrochiasmatic area |
| DTN | Dorsal tegmental nucleus | RE | Nucleus of reuniens |
| ECT | Ectorhinal area | RH | Rhomboid nucleus |
| ECU | External cuneate nucleus | RL | Rostral linear nucleus raphe |
| ENTl | Entorhinal area, lateral part | RM | Nucleus raphe magnus |
| ENTm | Entorhinal area, medial part | RN | Red nucleus |
| EP | Endopiriform nucleus | RO | Nucleus raphe obscurus |
| Eth | Ethmoid nucleus of the thalamus | RPA | Nucleus raphe pallidus |
| EW | Edinger-Westphal nucleus | RPF | Retroparafascicular nucleus |
| FC | Fasciola cinerea | RPO | Nucleus raphe pontis |
| FL | Flocculus | RR | Midbrain reticular nucleus, retrorubral area |
| FN | Fastigial nucleus | RSPagl | Retrosplenial area, lateral agranular part |
| FOTU | Folium-tuber vermis (VII) | RSPd | Retrosplenial area, dorsal part |
| FRP | Frontal pole | RSPv | Retrosplenial area, ventral part |
| FS | Fundus of striatum | RT | Reticular nucleus of the thalamus |
| GPe | Globus pallidus, external segment | SAG | Nucleus sagulum |
| GPi | Globus pallidus, internal segment | SBPV | Subparaventricular zone |
| GRN | Gigantocellular reticular nucleus | SCH | Suprachiasmatic nucleus |
| GU | Gustatory areas | SCm | Superior colliculus, motor related |
| HATA | Hippocampo-amygdalar transition area | SCO | Subcommissural organ |
| I5 | Intertrigeminal nucleus | SCs | Superior colliculus, superficial |
| IA | Intercalated amygdalar nucleus | SF | Septofimbrial nucleus |
| IAD | Interanterodorsal nucleus of the thalamus | SFO | Subfornical organ |
| IAM | Interanteromedial nucleus of the thalamus | SG | Supragenual nucleus |
| IC | Inferior colliculus | SGN | Suprageniculate nucleus |
| ICB | Infracerebellar nucleus | SH | Septohippocampal nucleus |
| IF | Interfascicular nucleus raphe | SI | Substantia innominata |
| IG | Induseum griseum | SIM | Simple lobule |
| IGL | Intergeniculate leaflet of the lateral geniculate complex | SLC | Subceruleus nucleus |
| III | Oculomotor nucleus | SLD | Sublaterodorsal nucleus |
| ILA | Infralimbic area | SMT | Submedial nucleus of the thalamus |
| IMD | Intermediodorsal nucleus of the thalamus | SNc | Substantia nigra, compact part |
| IntG | Intermediate geniculate nucleus | SNr | Substantia nigra, reticular part |
| IO | Inferior olivary complex | SO | Supraoptic nucleus |
| IP | Interposed nucleus | SOC | Superior olivary complex |
| IPN | Interpeduncular nucleus | SPA | Subparafascicular area |
| IRN | Intermediate reticular nucleus | SPF | Subparafascicular nucleus |
| ISN | Inferior salivatory nucleus | SPVC | Spinal nucleus of the trigeminal, caudal part |
| IV | Trochlear nucleus | SPVI | Spinal nucleus of the trigeminal, interpolar part |
| LA | Lateral Amygdalar nucleus | SPVO | Spinal nucleus of the trigeminal, oral part |
| LC | Locus ceruleus | SSp | Primary somatosensory area |
| LD | Lateral dorsal nucleus of thalamus | SSs | Supplemental somatosensory area |
| LDT | Laterodorsal tegmental nucleus | STN | Subthalamic nucleus |
| LGd | Dorsal part of the lateral geniculate complex | SUB | Subiculum |
| LGv | Ventral part of the lateral geniculate complex | SubG | Subgeniculate nucleus |
| LH | Lateral habenula | SUM | Supramammillary nucleus |
| LHA | Lateral hypothalamic area | SUT | Supratrigeminal nucleus |
| LIN | Linear nucleus of the medulla | TEa | Temporal association areas |
| LING | Lingula (I) | TM | Tuberomammillary nucleus |
| LM | Lateral mammillary nucleus | TR | Postpiriform transition area |
| LP | Lateral posterior nucleus of the thalamus | TRN | Tegmental reticular nucleus |
| LPO | Lateral preoptic area | TRS | Triangular nucleus of septum |
| LRN | Lateral reticular nucleus | TT | Taenia tecta |
| LS | Lateral septal nucleus | TU | Tuberal nucleus |
| LT | Lateral terminal nucleus of the accessory optic tract | UVU | Uvula (IX) |
| MA | Magnocellular nucleus | V | Motor nucleus of trigeminal |
| MA3 | Medial accesory oculomotor nucleus | VAL | Ventral anterior-lateral complex of the thalamus |
| MARN | Magnocellular reticular nucleus | VeCB | Vestibulocerebellar nucleus |
| MD | Mediodorsal nucleus of thalamus | VI | Abducens nucleus |
| MDRN | Medullary reticular nucleus | VII | Facial motor nucleus |
| MEA | Medial amygdalar nucleus | VISa | Anterior area |
| MEPO | Median preoptic nucleus | VISal | Anterolateral visual area |
| MEV | Midbrain trigeminal nucleus | VISam | Anteromedial visual area |
| MG | Medial geniculate complex | VISC | Visceral area |
| MH | Medial habenula | VISl | Lateral visual area |
| MM | Medial mammillary nucleus | VISli | Laterointermediate area |
| MOB | Main olfactory bulb | VISp | Primary visual area |
| MOp | Primary motor area | VISpl | Posterolateral visual area |
| MOs | Secondary motor area | VISpm | posteromedial visual area |
| MPN | Medial preoptic nucleus | VISpor | Postrhinal area |
| MPO | Medial preoptic area | VISrl | Rostrolateral area |
| MPT | Medial pretectal area | VLPO | Ventrolateral preoptic nucleus |
| MRN | Midbrain reticular nucleus | VM | Ventral medial nucleus of the thalamus |
| MS | Medial septal nucleus | VMH | Ventromedial hypothalamic nucleus |
| MT | Medial terminal nucleus of the accessory optic tract | VMPO | Ventromedial preoptic nucleus |
| NB | Nucleus of the brachium of the inferior colliculus | VNC | Vestibular nuclei |
| NDB | Diagonal band nucleus | VPL | Ventral posterolateral nucleus of the thalamus |
| NI | Nucleus incertus | VPLpc | Ventral posterolateral nucleus of the thalamus, parvicellular part |
| NLL | Nucleus of the lateral lemniscus | VPM | Ventral posteromedial nucleus of the thalamus |
| NLOT | Nucleus of the lateral olfactory tract | VPMpc | Ventral posteromedial nucleus of the thalamus, parvicellular part |
| NOD | Nodulus (X) | VTA | Ventral tegmental area |
| NOT | Nucleus of the optic tract | VTN | Ventral tegmental nucleus |
| NPC | Nucleus of the posterior commissure | x | Nucleus x |
| NTB | Nucleus of the trapezoid body | Xi | Xiphoid thalamic nucleus |
| NTS | Nucleus of the solitary tract | XII | Hypoglossal nucleus |
| OP | Olivary pretectal nucleus | y | Nucleus y |
| ORBl | Orbital area, lateral part | ZI | Zona incerta |
| ORBm | Orbital area, medial part |  |  |
