# Supplementary figures and images for: The Connectome and Chemo-Connectome Databases for Mice Brain Connection Analysis
Source: Front Neuroanat. 2022 Jun 9;16:886925. doi: 10.3389/fnana.2022.886925 (PMC9218099; doi:10.3389/fnana.2022.886925)

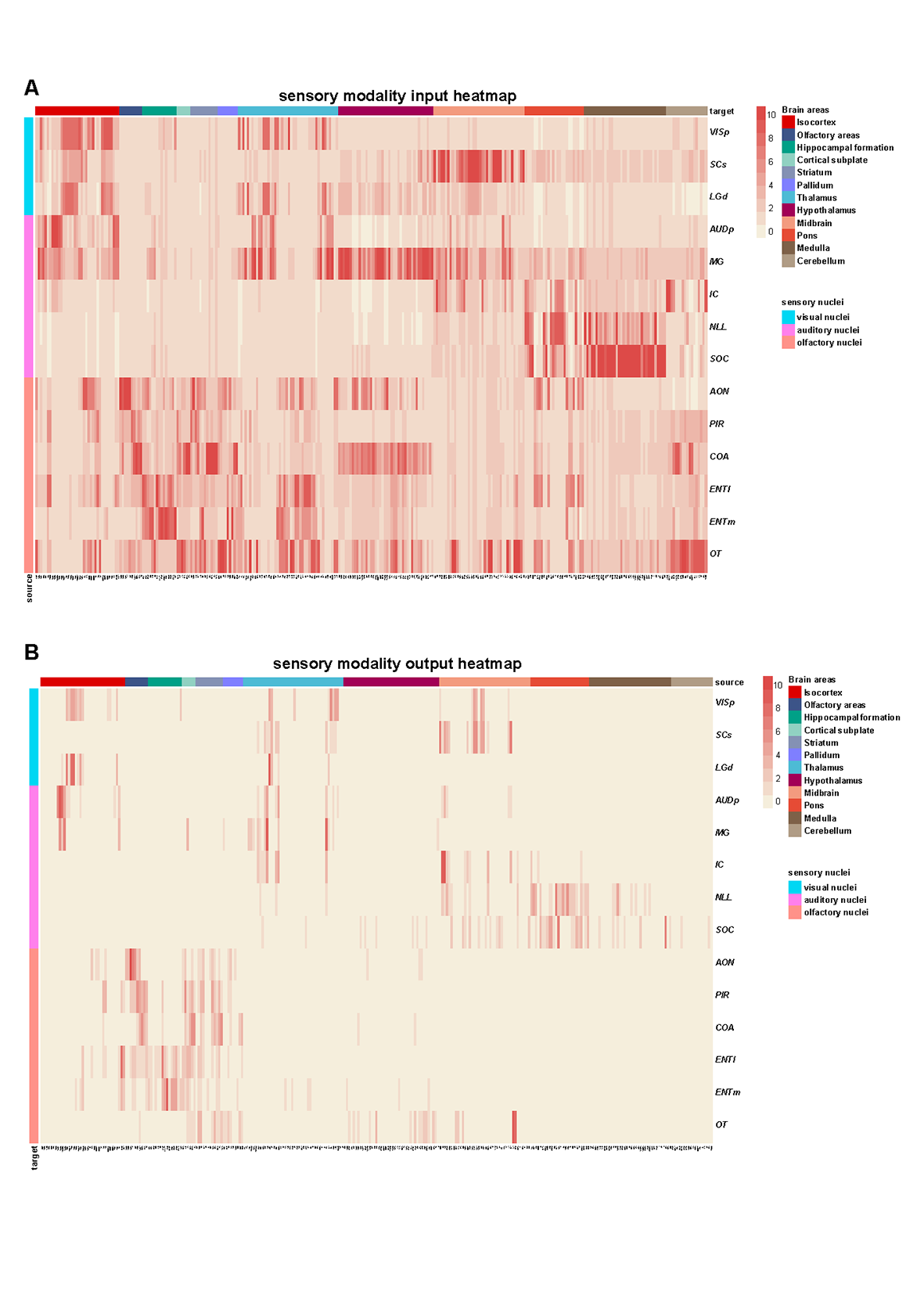

Supplement: Supplementary Figure 1 — The sensory modality connectivity heatmap. (A) The sensory modality input heatmap. (B) The sensory modality output heatmap. [file Image_1.TIFF]
